# Self-Adaptive Spectrum Analysis Based Bearing Fault Diagnosis

**DOI:** 10.3390/s18103312

**Published:** 2018-10-02

**Authors:** Jie Wu, Tang Tang, Ming Chen, Tianhao Hu

**Affiliations:** School of Mechanical Engineering, Tongji University, Shanghai 201804, China; 1710020@tongji.edu.cn (J.W.); chen.ming@tongji.edu.cn (M.C.); sirius.hu@tongji.edu.cn (T.H.)

**Keywords:** fault diagnosis, feature extraction, self-adaptive spectrum analysis, bearing

## Abstract

Bearings are critical parts of rotating machines, making bearing fault diagnosis based on signals a research hotspot through the ages. In real application scenarios, bearing signals are normally non-linear and unstable, and thus difficult to analyze in the time or frequency domain only. Meanwhile, fault feature vectors extracted conventionally with fixed dimensions may cause insufficiency or redundancy of diagnostic information and result in poor diagnostic performance. In this paper, Self-adaptive Spectrum Analysis (SSA) and a SSA-based diagnosis framework are proposed to solve these problems. Firstly, signals are decomposed into components with better analyzability. Then, SSA is developed to extract fault features adaptively and construct non-fixed dimension feature vectors. Finally, Support Vector Machine (SVM) is applied to classify different fault features. Data collected under different working conditions are selected for experiments. Results show that the diagnosis method based on the proposed diagnostic framework has better performance. In conclusion, combined with signal decomposition methods, the SSA method proposed in this paper achieves higher reliability and robustness than other tested feature extraction methods. Simultaneously, the diagnosis methods based on SSA achieve higher accuracy and stability under different working conditions with different sample division schemes.

## 1. Introduction

Bearings are critical parts in rotating machines and their health condition has a great impact on production. However, because of non-linear factors such as frictions, clearance and stiffness, vibration signals of bearings acquired in real application scenarios are characterized by non-linearity and instability which make bearing fault diagnosis difficult [1].

The general fault diagnosis process involves three main steps, namely signal acquisition and processing, fault feature extraction and fault feature classification [2]. Sensors are utilized to acquire signals with noises, and signal processing techniques are applied subsequently to improve the signal-to-noise ratio [3]. Particularly, ideal fault feature extraction can express the feature information of filtered signals comprehensively and efficiently, and it is the basis to produce an accurate fault feature classification. Therefore, a reasonable and efficient fault feature extraction plays an important role in fault diagnosis. Current fault extraction methods mainly include time domain, frequency domain and time-frequency domain analysis [4].

Time domain analysis is one of the earliest methods studied and applied. It calculates various statistical parameters in the time domain, for instance peak amplitude, kurtosis and skewness [5,6,7] to construct feature vectors. Frequency domain analysis transforms signals from the time domain into the frequency domain first, mainly focusing on Fourier Transform (FT) [8], then the periodical features, frequency features and distribution features of signals are extracted with methods such as cepstrum analysis and envelope spectrum analysis to construct feature vectors [9,10].

However, time domain or frequency domain analysis only extracts the information in the corresponding domain, resulting in the loss of information in the other domain. With in-depth study, time-frequency domain fault feature extraction methods were developed accordingly. They can extract both time and frequency information. They also have shown superiority for analyzing nonlinear and unstable signals.

As a typical time and frequency domain analysis method, Short Time Fourier Transform (STFT) [11] improves the analysis capability for unstable signals by introducing a fixed-width time window function. However, a fixed-width time window function in STFT cannot guarantee optimal time and frequency resolution simultaneously. The Wavelet Transform (WT) [12] introduces time and frequency scale coefficients to overcome the drawbacks of STFT. WT is based on the theory of inner product mapping and a reasonable basis function is the key to guarantee the effectiveness of WT. However, it is difficult to select a proper basis function. Therefore, to improve the adaptive analysis capability to signals, Empirical Mode Decomposition (EMD) [13] and Local Mean Decomposition (LMD) [14] methods were successively studied and applied. According to the local characters of signals themselves, EMD and LMD adaptively decompose a signal into various components which have better statistical characters for later analysis. Compared with each other, EMD is a mature tool for long-term study and usage, while LMD has an improved decomposition process and better decomposition results with physical explanations [15].

In recent years, EMD and LMD have been extensively studied and implemented. Mejia-Barron et al. [16] developed a method based on EMD to decompose signals and extract features, completing the fault diagnosis of winding faults. Saidi et al. [17] introduced a synthetical application of bi-spectrum and EMD to detect bearing faults. Cheng et al. [18] combined EEMD and entropy fusion to extract fault features for planetary gearboxes, and furthermore implemented fault diagnosis successfully. Yi et al. [19] also utilized EEMD to pre-process signals for further fault diagnosis for bearings. Liu and Han et al. [20] applied LMD and multi-scale entropy methods to extract fault features and analyzed faults successfully. Yang et al. [21] proposed an ensemble local mean decomposition method and applied it in rub-impact fault diagnosis for rotor systems. Han and Pan et al. [22] integrated LMD, sample entropy and energy ratio to process vibration signals and realized the fault feature extraction and fault diagnosis in rolling element bearings. Yasir and Koh et al. [23] adopted LMD and multi-scale permutation entropy and realized bearing fault diagnosis. Guo et al. [24] studied an improved fault diagnosis method for gearbox combining LMD and a synchrosqueezing transform.

Fault feature classification is implemented after fault feature extraction. Nowadays, shallow machine learning methods are extensively utilized to solve the classification problem. Support Vector Machine, Artificial Neural Network and Fuzzy Logical System are widely applied in condition monitoring and fault diagnosis [25]. Particularly, SVM is based on statistics and minimum theory of structured risk, and it has better classification performance when dealing with the practical problems of a small amount of and non-linear samples. To solve the multi-class classification problems, based on SVM, Cherkassky [26] proposed a one-against-all (oaa) strategy in his studies, transforming a N-class classification problem into *N* binary classification problems. Also, Kressel [27] used a method to transform a N-class classification problem into N(N−1)/2 binary classification problems, namely the one-against-one (oao) strategy. Wu et al. [28] adopted SVM to diagnosis via analyzing the full-spectrum to extract fault features. Saimurugan et al. [29] improved the diagnosis performance by integrating SVM and avdecision tree. Santos et al. [30] selected SVM for classification in wind turbine fault diagnosis with several trails of different kernels.

Currently, researchers all over the world have carried out extensive studies on bearing fault diagnosis. To our best knowledge, fault diagnosis methods still need further study, although various solutions have been investigated from different aspects. The main problems to be solved in this paper are summarized as follows:

(1) Vibration signals acquired in real application scenarios are non-linear and unstable and their statistical characters are time-varying. Hence, it is difficult to extract effective and comprehensive fault features only in the time-domain or in frequency-domain.
(2) Conventional fault feature extraction methods take the overall characteristics of signals into account via calculating statistical parameters to construct feature vectors with fixed dimensions, however, local detailed characteristics are neglected. Therefore, fault information contained in vectors may be insufficient or redundant in different working conditions because vectors have a fixed dimension, consequently leading to lower reliability and robustness of fault feature extraction. Meanwhile, data-driven classifiers are sensitive to classification features and minor changes in classification features may result in performance reduction [31].

In order to improve the fault diagnosis performance, in this paper, SSA is proposed to adaptively extract fault features and construct unfixed-dimension feature vectors according to local characters of signals. Then, SSA is implemented under the designed framework. Signals are decomposed firstly to obtain components with better analyzability, LMD and EEMD are both utilized to decompose signals into different components from different analysis aspects. SSA is utilized to extract fault features adaptively and feature vectors with non-fixed dimensions are constructed subsequently. Finally, SVM is selected to classify the fault features considering its inherent advantages to small amount train samples.

## 2. Methodology

### 2.1. Self-Adaptive Spectrum Analysis

Aiming at solving the problem that conventional feature extraction methods neglect local details of signal and fault information may be redundant or insufficient because of fixed-dimension feature vectors, Self-adaptive Spectrum Analysis (SSA) is proposed. With the SSA method, unfixed-dimension feature vectors are constructed by extracting the local characteristics of signals adaptively.

At first, a number of signals corresponding to different categories of fault types are selected. To implement SSA method efficiently, Fast Fourier Transform (FFT) is used to transform the signals into frequency domain to get corresponding spectrums for better readability. Then an overall frequency-window is set to all spectrums according to the fluctuation in spectrums, and local feature information inside the frequency-window is extracted to construct feature vectors.

In order to implement the proposed SSA, some definitions are given:

**Definition**  **1.** *Differential frequency*$f_{z}$.

$f_{z}$ is the minimum frequency unit in SSA. Normally, feature information is extracted at points corresponding to $nf_{z}$ (*n* = 1, 2, 3,…), where $f_{z}$ is calculated as follows:

Firstly, in each spectrum, the maximum amplitude and corresponding frequency value are found. All the frequency values are denoted as $f_{1}$, $f_{2}$, $f_{3}$, *…*, $f_{m}$, where *m* means the sequence number of signals. More than two fault categories must be included within the selected signals.

Secondly, the frequency values are arranged into different vectors according to the categories of samples; vectors are denoted as:

(1) $$\;v_{i}=\left[f_{(i-1)m/k+1},f_{\left(i-1\right)m/k+2},\ldots ,f_{\left(i-1\right)m/k+m/k}\right]\;$$

where *k* means *k* kinds of faults， i=[1, 2,…k]. Here we assume that different categories have the same amount of signals. Then, the average values of all elements in each vector are figured out and denoted as ${\bar{v}}_{1}$, ${\bar{v}}_{2}$,…, ${\bar{v}}_{k}$, respectively, then a vector $f=\left[{\bar{v}}_{1},{\bar{v}}_{2}\ldots {\bar{v}}_{k}\right]$ is constructed.

Thirdly, minimum frequency value $f_{\min}$ and the maximum frequency value $f_{\max}$ are selected in vector f. Then, two neighboring frequency values are also selected in f, between which there is the maximum value among the differences between every two neighboring frequencies, the lower frequency is denoted as $f_{\mathrm{low}}$ and the higher one is denoted as $f_{\mathrm{high}}$.

Finally, $f_{\min}$, $f_{\max}$, $f_{\mathrm{low}}$, $f_{\mathrm{high}}$ are arranged in ascending order, and absolute values $f_{\mathrm{diff}}$ of differences between every neighboring two frequencies are calculated. The minimum non-zero $f_{\mathrm{diff}}$ value is picked to be the value of $f_{z}$:

(2) $$\;f_{z}=min\left(f_{\mathrm{diff}}\right)\;$$

**Definition**  **2.** *Frequency Window*$W=\;[f_{l},f_{r}]$.

The frequency window is a specific frequency section for extracting feature information, $f_{l}$ is the left boundary while $f_{r}$ is the right boundary. Frequency window is determined with fixed boundaries, and feature information is extracted inner the window. Boundaries are calculated as follows:

(3) $$\;f_{l}=floor\left(\frac{f_{\min}}{f_{z}}\right)*f_{z}\;$$

(4) $$\;f_{r}=ceil\left(\frac{f_{\max}}{f_{z}}\right)*f_{z}\;$$

where floor(*) is a round down function, ceil(*) is a round up function.

**Definition**  **3.** *Tolerance*μ.

Tolerance μ denotes that in a section which is centered with $nf_{z}$, μ is taken as the semidiameter to determine the searching section ($nf_{z}-\mu$, $nf_{z}+\mu \;$], and the maximum amplitude value corresponding to a frequency within this section can be regarded as the amplitude value to $nf_{z}$. μ is calculated as follows:

(5) $$\;\mu =floor\left(\frac{f_{z}}{2}\right)\;$$

**Definition**  **4.** *Peak value ratio coefficient*h.

h denotes the degree of peak amplitude value. It is utilized to judge whether the amplitude value is normal or not and all h construct fault feature vectors. h is calculated as follows:

Firstly, average value of all the amplitude values in frequency window [$f_{l},f_{r}$] is calculated, denoted as $A_{\mathrm{ave}}$, also the maximum amplitude value in section ($nf_{z}-\mu$, $nf_{z}+\mu \;$] is selected and denoted as $A_{\max}$. Finrfally, h can be calculated as follows:

(6) $$\;h=\frac{A_{max}}{A_{ave}}\;$$

**Figure 1:**
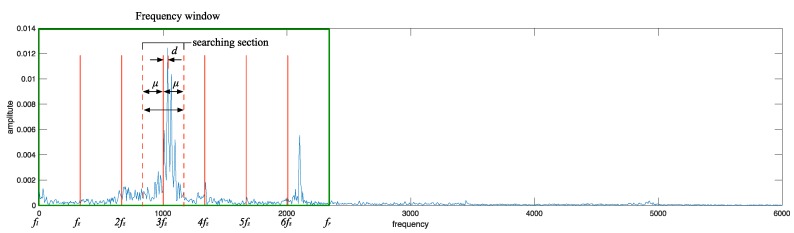
Sketch map of parameters in adaptive spectrum analysis.

Figure 1 gives a description of the definitions mentioned above.

Combined with Figure 1, SSA is implemented on each spectrum as follows:

(1) Calculating values of differential frequency $f_{z}$ and boundaries $f_{l}$, $f_{r}$, frequency window W is determined;
(2) Calculating all the $nf_{z}$ values, taking μ as side intervals to determine different searching sections;
(3) Selecting the maximum amplitude in each searching section and corresponding frequency value, calculating the absolute frequency interval d between this frequency value and section center $nf_{z}$, also, h are calculated, frequency interval vector $D=\left[d_{1},d_{2},d_{3}\ldots \;d_{n}\right]$, Peak value ratio coefficient vector $\;H=\left[h_{1},h_{2},h_{3}\ldots \;h_{n}\right]$;
(4) Setting a threshold value $h_{t}$ for h, and $h_{t}$ could be optimized automatically by the overall accuracy. $h_{t}$ is used to judge if an anomaly exists in sections. When $h>h_{t}$, the corresponding section is regarded as an abnormal one;
(5) If an anomaly is found, figuring out whether all the frequency values corresponding to abnormal sections are on the same side of $nf_{z}$ (*n* = 1, 2, 3,…) along the frequency axis simultaneously. If they are on the same side, selecting a minimum d in D, and shifting the spectrum to the opposite direction by d. Subsequently, repeating steps 1 to 3. While, if they are not on the same side, skip steps 5 and 6;
(6) H is taken as the fault feature vector extracted from the spectrum.

### 2.2. Framework Construction of Fault Diagnosis

**Figure 2:**
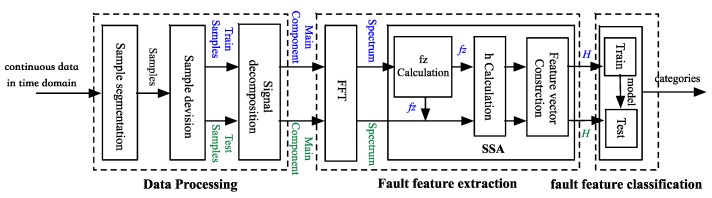
SSA-based diagnostic framework.

The overall framework construction of the proposed fault diagnosis method based on SSA in our research is shown in Figure 2.

The proposed fault diagnosis method includes three parts, namely data processing, fault feature extraction and fault feature classification.

#### 2.2.1. Data Processing

**Figure 3:**
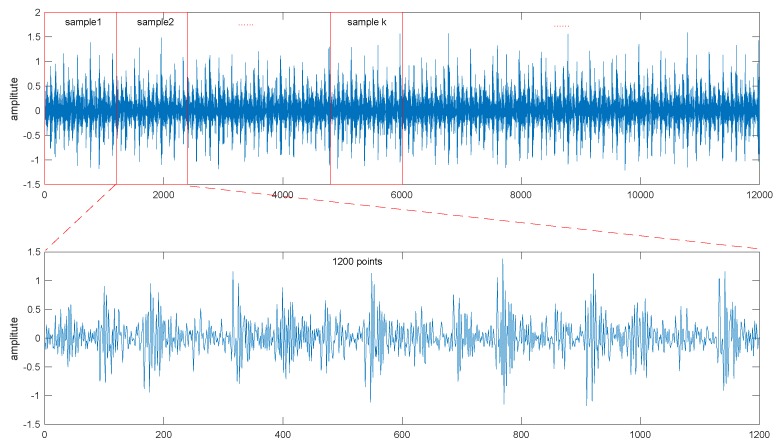
Segmentation of samples.

As shown in Figure 3, a signal segment containing 120,000 points is selected, then it is segmented into 100 parts with a same length. In total, 100 samples are extracted from one signal segment. Therewith, 100 samples are separated into a training sample set and a test sample set.

Each sample is decomposed into a set of components with better analyzability with a time-frequency analysis method, LMD and EEMD are two commonly used ones. The very first component in each set of components is chosen to extract fault features because they accumulate the main part of the energy.

#### 2.2.2. Fault Feature Extraction

FFT is utilized to transform the decomposed component into the frequency domain, and then SSA is implemented to extract fault features. First the components of the training samples are selected to calculate $f_{z}$, and $f_{z}$ is utilized for both the training samples and test samples to extract fault features. 

#### 2.2.3. Fault Feature Classification

Fault feature vectors are classified into different fault patterns. Vectors extracted from the training samples are utilized to train the classification model and parameters are tuned to optimize the model. Here, SVM is selected because of its better performance in classification with small samples. Eventually, categories are output with the well-trained model.

### 2.3. Experiment Preparation

#### 2.3.1. Data Selection and Processing

Vibration signals acquired from bearings are utilized for validation. In this paper, selected bearing data published by Case Western Reverse University were used [32]. Single point faults are introduced to the test bearings on different parts (ball, inner race and outer race) to simulate different kinds of faults. Vibration signals of different kinds of faults with different failure degrees are collected under different loads to construct the experimental data set.

The data set consisted of vibration data collected on SKF bearings, and the sampling frequency is 12 kHz. Twelve kinds of combinations under four kinds of loads (0, 1, 2 and 3 hp) and three kinds of failure degrees (0.007, 0.014 and 0.021 inch) form 12 different working conditions.

Under each working condition, four kinds of fault mode (normal, ball fault, inner race fault and outer race fault) are simulated, and four time-varying signals corresponding to the faults are collected, respectively. Each signal is processed with the proposed method given in Figure 3 to extract 100 samples, and 100 feature vectors are subsequently constructed. Eventually, 400 feature vectors are determined under every working condition.

#### 2.3.2. Parameter Determination

Parameters corresponding to decomposition methods, fault feature extraction process and fault feature classification modeling process are determined as follows:

Parameters to be determined in signal decomposition methods:

(1) In LMD, parameters are determined according to reference [33];
(2) In EEMD, parameters are determined according to reference [34];

Parameters to be determined in SSA method:

(1) $f_{z}$, differential frequency value is calculated according to Equation (2);
(2) $f_{l}$ , left boundary value is calculated according to Equation (3);
(3) $f_{r}$, right boundary value is calculated according to Equation (4);
(4) μ, tolerance value is calculated according to Equation (5);
(5) h, peak value coefficient ratio is calculated according to Equation (6);
(6) $h_{t}$, the minimum value in vector H is selected as the threshold value of h;

Parameters to be determined in pattern recognition method:

(1) In SVM, cost *c* is a basic parameter while *g* is a specific one in RBF kernel. In this paper, Grid search [35] is applied and overall accuracy is taken into consideration to tune the two parameters.

## 3. Experiments and Results

### Experiment Results and Analysis

In this subsection, a simulated signal x(t) is utilized to evaluate the effectivity of decomposition methods [33].  x(t) consists of two superimposed component signals: $x\left(t\right)=\left(1+0.5cos\left(9\pi t\right)\right)cos\left(200\pi t+2cos\left(10\pi t\right)\right)+3cos\left(20\pi t^{2}+6\pi t\right)\;t\in \left[0,1\right]$

**Figure 4:**
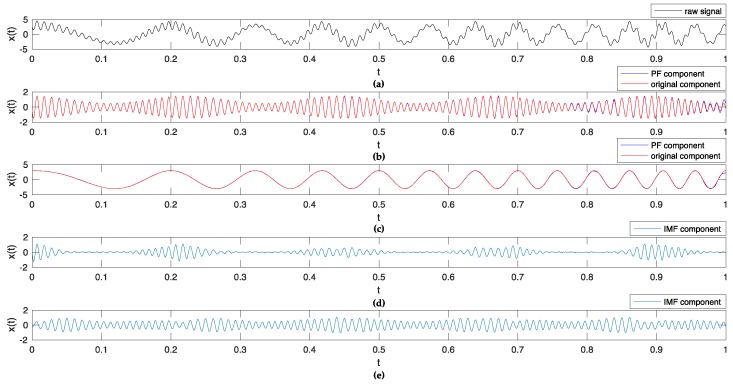
Results of decomposition for simulated signal.

The LMD and EEMD methods are used to decompose the signal. Figure 4 illustrates the results of the decomposition.

Figure 4a shows the oscillograph of the simulated signal. In Figure 4b,c, the oscillographs in red are two original components of the raw simulated signal, and the ones in blue are the Product Function (PF) components extracted with the LMD method. Obviously, the original components and extracted PF components have a high similarity except for several end points on the right. In Figure 4d,e, the oscillographs in blue are the first two Intrinsic Mode Function (IMF) components extracted with the EEMD method; both of them have less similarity with the original ones. These results prove that LMD adopted in the research can effectively decompose the raw signal into PF components which have physical significance, and EEMD can decompose the raw signal into IMF components by another mechanism [18].

Four experimental sets are designed combining two different signal decomposition methods: two different feature extraction methods and a fault feature classification method. The four experimental sets are arranged as shown in Table 1. LMD and EEMD are utilized to decompose the signals. The fault feature extraction methods include the proposed SSA and the combination of Sample Entropy (SE) and Energy Ratio (ER) [36], and the LIBSVM [37] software package is selected to implement the pattern classification.

In each experiment set, 12 kinds of working conditions (a working condition is denoted as a load-fault five kinds of sample division scheme are tested (a sample division scheme is denoted as: number of training samples in 100 samples to every fault/number of test samples in 100 samples to every fault, for example 5/95, 10/90, 20/80, 40/60, 60/40), with each scheme, 10 independent experiments are repeated. Ultimately, 2400 experiments are carried out in total within the four experiment sets. Table 2 shows the $f_{z}$ values and dimensions of feature vectors under 12 working conditions with sample division schemes of 5/95 and 60/40, respectively, in experimental set 1.

The results illustrate that when the working condition or division scheme changes, the differential frequency $f_{z}$ value and dimension value of the feature vectors change accordingly.

Without considering sample division schemes, the overall diagnostic capability of proposed model is evaluated. The average values and variance of accuracy values to all independent experiments (50 times) under each working condition are listed in Table 3 and Table 4.

**Figure 5:**
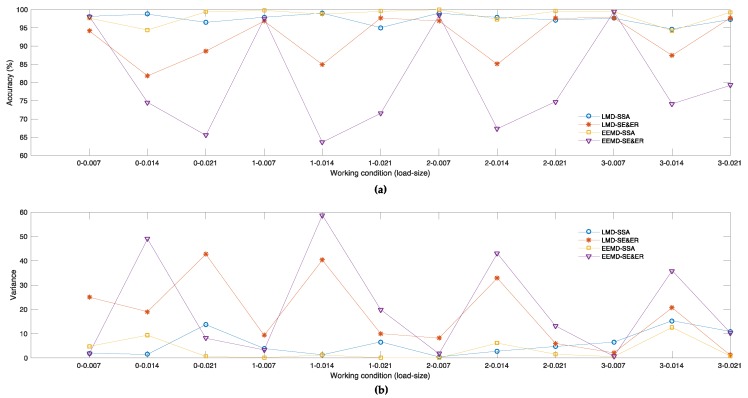
(**a**) Average diagnostic accuracy of all independent experiments corresponding to 12 different working conditions respectively in 1st–4th experimental sets. (**b**) Variance of diagnostic accuracy of all independent experiments corresponding to 12 different working conditions respectively in 1st–4th experimental set.

Figure 5a,b transforms Table 3 and Table 4 in graphic ways, respectively.

Table 3 and Figure 5a show that Set 3 achieves the best average accuracies under six kinds of working conditions and Set 1 achieves the best under five kinds of working conditions, while Set 4 only ranks the first place under one kind of working conditions. Overall, the average accuracies of Set 1 and Set 3 are 97.42% and 98.27%, maintaining a higher level, yet the average accuracies of Set 2 and Set 4 are only 92.21% and 80.36%, being especially worse in severe failure situations. Meanwhile, the variance of average accuracies under different working conditions of Set 1 is 1.99, and the numbers of Set 2, Set 3 and Set 4 are 37.84, 4.08 and 195.72, respectively. Obviously, the statistics of Set 1 and Set 3 indicate better performances than Set 2 and Set 4.

Table 4 and Figure 5b show that Set 3 obtains the smallest variances under nine working conditions and Set 1 is the least under to kinds of working conditions, while Set 4 only performs best under one working condition. Clearly, the average value of variance values under 12 different working conditions for Set 1, Set 2, Set 3 and Set 4 are 5.81, 18.12, 3.17 and 20.52, respectively. Set 1 and Set 3 outperform Set 2 and Set 4. Meanwhile the values of the variances of Set 2 and Set 4 show obvious fluctuation under severe failure conditions.

As a matter of fact, to simulate a situation that labeled data are rare in real application scenarios, a small amount of samples division scheme is tested. Average values and variances of the accuracy values of all independent experiments (10 times) under each working condition with a (5/95) sample division scheme are shown in Table 5 and Table 6.

**Figure 6:**
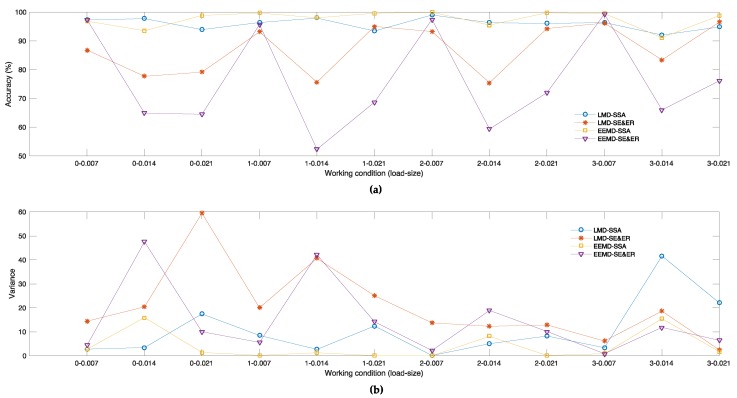
(**a**) Average diagnostic accuracy to all independent experiments corresponding to 12 different working conditions respectively with the scheme of (5/95) in the 1st–4th experimental sets. (**b**) Variance of diagnostic accuracy to all independent experiments corresponding to 12 different working conditions, respectively, with the scheme of (5/95) in the 1st–4th experimental sets.

Figure 6a,b also illustrates the results of Table 5 and Table 6 in graphic ways, respectively.

Table 5 and Figure 6a show that Set 3 achieves the best average accuracies under eight kinds of working conditions and Set 1 achieves the best under two kinds of working conditions, while Set 4 only ranks the first place under two kinds of working conditions. Overall, the average accuracies of Set 1 and Set 3 are 95.94% and 97.59%, still maintaining a high level with slight decreases compared to the overall average accuracies, yet the average accuracies of Set 2 and Set 4 are only 87.22% and 76.15%, being especially worse in severe failure situations, and showing sharp decreases compared to overall average accuracies. Meanwhile, the variance of average accuracies under different working conditions of Set 1 and Set 3 are 10.65 and 4.00, and the numbers for Set 2 and Set 4 are 20.51 and 15.4, respectively. Obviously, the results in Set 1 and Set 3 are better than the results in Set 2 and Set 4.

Table 6 and Figure 6b show that the variance values of 50 independent experiments in Set 3 under each working condition maintain a steady low level and the mean value of 12 values is 4.00. While in Set 1, variance values appear obvious fluctuation under 3–0.014 only, and the mean value is 10.65; values in Set 2 and Set 4 fluctuate wildly and the mean values are 20.51 and 14.54, respectively.

**Figure 7:**
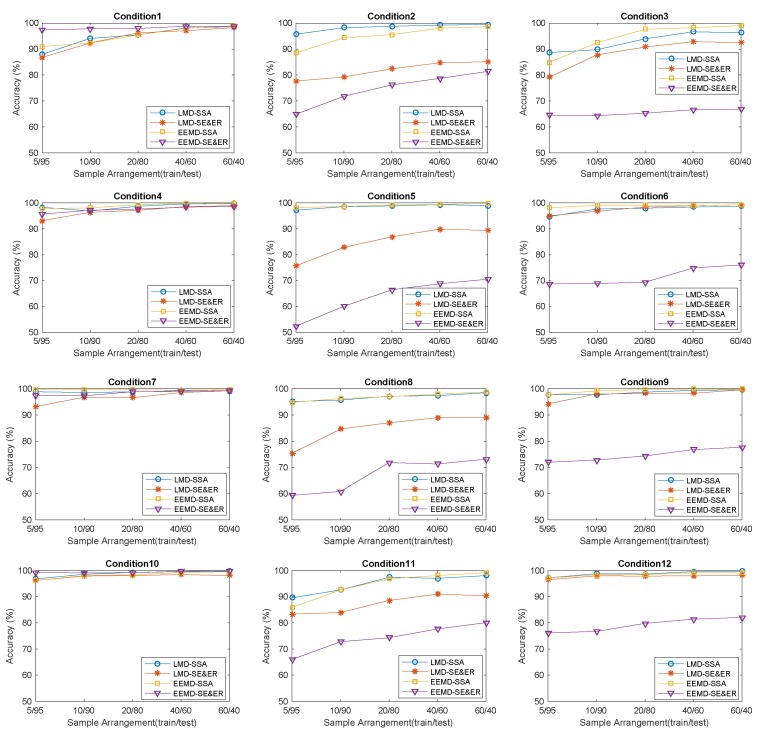
Average diagnostic accuracy to all independent experiments corresponding to 12 different working conditions, respectively, with different schemes in the 1st–4th experimental sets.

The convergence performance with the increase of the amount of training samples is also a key indicator to evaluate a model. Experiments are conducted under different working conditions with different sample division schemes, also, mean value and variance of accuracy values of all independent experiments (10 times) are calculated and listed in Table 7. Figure 7 transforms Table 7 into diagrams.

In Table 7, 60 comparisons are conducted under different working conditions with different sample division schemes, and the results show that Set 1 and Set 3 have better performance in average accuracy with 56 comparisons out of 60, while Set 2 or Set 4 only get higher accuracies under the working condition of 0–0.007 with four kinds of schemes. As shown in Figure 7, with the increase of the number of training samples, the average accuracies of Set 1 and Set 3 obviously converge toward the highest value faster than Set 2 and Set 4.

Considering all the results comprehensively, further analysis is carried out. LMD and EEMD can decompose nonlinear and unstable signals into a set of components in the time domain, and these components have better analyzability. The proposed SSA method can adaptively extract feature information according to local characteristics, and construct unfixed-dimension fault feature vectors, and it is proved to have better efficiency and robustness. SSA-based fault diagnosis methods can obtain higher accuracies under different working conditions with different sample division schemes in most comparisons (56/60), and the accuracies show less fluctuation between different conditions. With the increasing number of samples, the accuracies achieved with the SSA-based method converge towards the highest values faster. Especially with a small sample division scheme (5/95), the results have shown that methods based on SSA still maintain high accuracy and stability and they are proved specially suitable for practical application in scenarios with small amounts of training samples.

## 4. Conclusions

To improve the fault extraction performance, SSA is proposed in this paper. Combined with signal decomposition methods, SSA extracts fault features from non-linear and unstable signals effectively, then fault features are classified with SVM. Bearing data under 12 different working conditions obtained from CWRU are utilized to evaluate the diagnosis methods. The conclusions may be summarized as follows:

1. SSA extracts fault features and constructs unfixed-dimension vectors adaptively, it has reduced the side effects caused by information insufficiency and redundancy. Moreover, SSA has higher efficiency and robustness in fault extraction.
2. Fault diagnosis methods based on SSA can achieve higher accuracies and stability than other methods under the same proposed framework and with an increased number of training samples, the accuracies achieved with the SSA-based method converge to the highest value faster.
3. Especially, with a small amount training samples, the SSA-based method still provides high accuracy with more obvious superiority in accuracy and stability, therefore they have the potential to be implemented in real application scenarios.

## 5. Future Lines of Work

In recent years, deep learning has been adopted gradually in fault diagnosis. It can extract fault features automatically because of its multi-layer structure, this characteristic can improve the feature extraction further. At the same time, transfer learning [38] has achieved great success in many fields. Its generalization capability can be also utilized in fault diagnosis to promote the diagnostic theories to applications. Therefore, our future work will be focused on the study of implementation of the combination of deep learning and transfer learning in fault diagnosis.

## Figures and Tables

**Table 1:** Arrangement of experiments.

Experiment Set	Signal Decomposition	Fault Feature Extraction	Fault Feature Classification
Set 1	LMD	SSA	SVM
Set 2	LMD	SE&ER	SVM
Set 3	EEMD	SSA	SVM
Set 4	EEMD	SE&ER	SVM

**Table 2:** Values of differential frequency and dimensions of character vectors.

Division Scheme	Working Condition	0–0.007	0–0.014	0–0.021	1–0.007	1–0.014	1–0.021
5/95	$f_{z}$ (Hz)	94	234	369	533	217	486
	Dimension	37	15	9	7	16	7
60/40	$f_{z}$ (Hz)	229	334	375	451	176	504
	Dimension	16	11	9	8	20	7
**Division Scheme**	**Working Condition**	**2–0.007**	**2–0.014**	**2–0.021**	**3–0.007**	**3–0.014**	**3–0.021**
5/95	$f_{z}$ (Hz)	563	234	656	586	580	574
	Dimension	7	15	6	6	6	6
60/40	$f_{z}$ (Hz)	463	240	598	580	440	568
	Dimension	8	15	6	6	8	6

**Table 3:** Average diagnostic accuracy of all independent experiments corresponding to 12 different working conditions respectively in 1st–4th experiment sets.

Working Condition	Set 1	Set 2	Set 3	Set 4
0–0.007	98.14	94.15	97.65	98.12
0–0.014	98.75	81.83	94.41	74.59
0–0.021	96.48	88.61	99.36	65.52
1–0.007	97.91	96.85	99.80	97.51
1–0.014	99.02	84.93	98.75	63.61
1–0.021	95.07	97.69	99.53	71.55
2–0.007	99.03	96.90	99.97	98.42
2–0.014	97.89	85.03	97.36	67.33
2–0.021	97.18	97.70	99.54	74.77
3–0.007	97.62	97.74	99.42	99.46
3–0.014	94.67	87.44	94.20	74.16
3–0.021	97.26	97.71	99.26	79.23
Average	97.42	92.21	98.27	80.36

**Table 4:** Variances of diagnostic accuracy of all independent experiments corresponding to 12 different working conditions respectively in 1st–4th experiment sets.

Working Condition	Set 1	Set 2	Set 3	Set 4
0–0.007	1.94	25.00	4.85	1.93
0–0.014	1.50	19.08	9.39	49.01
0–0.021	13.75	42.70	0.63	8.15
1–0.007	3.90	9.32	0.13	3.32
1–0.014	1.36	40.30	1.27	58.80
1–0.021	6.32	10.01	0.10	19.94
2–0.007	0.49	8.23	0.01	1.74
2–0.014	2.82	32.84	6.12	43.05
2–0.021	4.77	6.04	1.55	13.25
3–0.007	6.55	2.12	0.61	0.71
3–0.014	15.34	20.66	12.60	35.93
3–0.021	10.95	1.16	0.82	10.36
Average	5.81	18.12	3.17	20.52

**Table 5:** Average diagnostic accuracy to all independent experiments corresponding to 12 different working conditions respectively with the scheme (5/95) in 1st–4th experiment sets.

Working Condition	Set 1	Set 2	Set 3	Set 4
0–0.007	97.26	86.82	96.84	97.37
0–0.014	97.74	77.68	93.53	64.89
0–0.021	93.92	79.13	98.84	64.50
1–0.007	96.39	93.16	99.68	95.68
1–0.014	97.95	75.68	98.00	52.34
1–0.021	93.45	95.03	99.61	68.68
2–0.007	98.97	93.26	99.97	97.45
2–0.014	96.32	75.47	95.53	59.37
2–0.021	96.03	94.32	99.74	72.00
3–0.007	96.39	96.18	99.45	99.34
3–0.014	92.05	83.37	91.13	66.00
3–0.021	94.82	96.50	98.79	76.16
Average	95.94	87.22	97.59	76.15

**Table 6:** Variances of diagnostic accuracy to all independent experiments corresponding to 12 different working conditions respectively with the scheme of (5/95) in 1st–4th experiment sets.

Working Condition	Set 1	Set 2	Set 3	Set 4
0–0.007	2.80	14.43	2.74	4.45
0–0.014	3.43	20.42	15.94	47.56
0–0.021	17.47	59.47	1.40	10.03
1–0.007	8.50	20.01	0.21	5.60
1–0.014	2.67	40.65	1.28	42.14
1–0.021	12.41	25.03	0.07	14.24
2–0.007	0.16	13.75	0.01	2.22
2–0.014	5.08	12.28	8.14	18.92
2–0.021	8.26	12.88	0.15	10.14
3–0.007	3.40	6.16	0.90	0.87
3–0.014	41.64	18.63	15.50	11.85
3–0.021	22.03	2.46	1.68	6.45
Average	10.65	20.51	4.00	14.54

**Table 7:** Average diagnostic accuracy to all independent experiments corresponding to 12 different working conditions respectively with different schemes in 1st–4th experiment sets.

	0–0.007					0–0.014				
	5/95	10/90	20/80	40/60	60/40	5/95	10/90	20/80	40/60	60/40
Set 1	97.26	97.31	98.28	98.96	98.88	97.74	98.33	99	99.13	99.56
Set 2	86.82	92.36	96.25	97.08	98.25	77.68	79.22	82.41	84.71	85.13
Set 3	96.84	95.92	97.22	98.79	99.5	93.53	93.72	95.34	94.63	94.81
Set 4	97.37	97.83	97.97	98.79	98.63	64.89	71.78	76.19	78.71	81.38
	**0–0.021**					**1–0.007**				
	**5/95**	**10/90**	**20/80**	**40/60**	**60/40**	**5/95**	**10/90**	**20/80**	**40/60**	**60/40**
Set 1	93.92	94.97	97.06	98.79	97.69	96.39	98.03	98.19	98.5	98.44
Set 2	79.13	87.81	90.78	92.83	92.5	93.16	96.39	97.16	98.63	98.94
Set 3	98.84	99.64	99.66	99.33	99.31	99.68	99.81	99.84	99.92	99.75
Set 4	64.5	64.36	65.28	66.63	66.81	95.68	97.19	97.63	98.38	98.69
	**1–0.014**					**1–0.021**				
	**5/95**	**10/90**	**20/80**	**40/60**	**60/40**	**5/95**	**10/90**	**20/80**	**40/60**	**60/40**
Set 1	97.95	99.17	98.91	99.58	99.5	93.45	94.81	96	95.17	95.94
Set 2	75.68	82.83	86.91	89.88	89.38	95.03	96.81	98.41	98.92	99.31
Set 3	98	98.08	98.94	99.17	99.56	99.61	99.53	99.44	99.38	99.69
Set 4	52.34	60.03	66.44	68.75	70.5	68.68	68.86	69.34	74.88	76
	**2–0.007**					**2–0.014**				
	**5/95**	**10/90**	**20/80**	**40/60**	**60/40**	**5/95**	**10/90**	**20/80**	**40/60**	**60/40**
Set 1	98.97	99.25	98.47	99.46	99	96.32	97.42	98.34	98.79	98.56
Set 2	93.26	96.78	96.63	98.58	99.25	75.47	84.67	87.06	89	88.94
Set 3	99.97	99.97	99.97	99.96	100	95.53	96.72	97.25	98.88	98.44
Set 4	97.45	97.36	98.91	99.08	99.31	59.37	60.86	71.88	71.33	73.19
	**2–0.021**					**3–0.007**				
	**5/95**	**10/90**	**20/80**	**40/60**	**60/40**	**5/95**	**10/90**	**20/80**	**40/60**	**60/40**
Set 1	96.03	96.86	97.25	97.13	98.63	96.39	95.5	98.41	98.79	99
Set 2	94.32	98.03	98.22	98.33	99.63	96.18	97.89	98.13	98.46	98.06
Set 3	99.74	98.53	99.66	99.96	99.81	99.45	99.31	98.97	99.54	99.81
Set 4	72	72.86	74.41	76.83	77.75	99.34	99.08	99.22	99.71	99.94
	**3–0.014**					**3–0.021**				
	**5/95**	**10/90**	**20/80**	**40/60**	**60/40**	**5/95**	**10/90**	**20/80**	**40/60**	**60/40**
Set 1	92.05	93.44	94.75	96.71	96.38	94.82	96.03	97.53	99.17	98.75
Set 2	83.37	83.92	88.59	90.96	90.38	96.5	97.97	97.91	98	98.19
Set 3	91.13	93.14	95.06	94.79	96.88	98.79	99.25	98.94	99.63	99.69
Set 4	66	72.78	74.28	77.67	80.06	76.16	76.72	79.78	81.42	82.06
